# Too Much of a Good Thing? Exercise Dependence in Endurance Athletes: Relationships with Personal and Social Resources

**DOI:** 10.3390/ijerph18062966

**Published:** 2021-03-14

**Authors:** Zsuzsanna Zimanyi, Wanja Wolff, Julia Schüler

**Affiliations:** 1 Department of Sport Science, Sport Psychology, University of Konstanz, 78464 Konstanz, Germany; zimanyizsuzsanna@gmail.com (Z.Z.); wanja.wolff@uni-konstanz.de (W.W.); 2 Institute of Educational Science, Educational Psychology, University of Bern, 3012 Bern, Switzerland

**Keywords:** exercise dependence, endurance sports, self-control, self-concordance, social support

## Abstract

(1) Background: A large body of research has examined the positive effects of physical activity on physical and mental health. However, for some, excessive exercise can develop into an addiction that is detrimental to their health. In the present study, we examine potential personal (self-control, self-concordance) and social (social support) resources that we assume to be related to exercise dependence. (2) Methods: One hundred and forty athletes from different endurance sports participated in an online survey. Exercise dependence, self-control, self-concordance, and social support were assessed using questionnaires that are well-established in health and sport psychology. Additionally, further sport-relevant and demographic variables were assessed. (3) Results: Correlational analyses supported our hypotheses that exercise dependence is negatively correlated with the personal resources trait, state self-control, and self-concordance. Social support, however, was not significantly correlated with exercise dependence. Furthermore, the results of a mediation analysis revealed that the relationship between both personal traits (self-control, self-concordance) and exercise dependence was mediated by state self-control. (4) Conclusions: Our results indicate that trait self-control and self-concordance might be important personal resources that protect against exercise dependence by making state self-control available.

## 1. Introduction

A large body of research has shown that physical activity has a positive effect on physical and mental health [1]. Recommendations for the minimum level of physical activity that is needed for beneficial health effects are given by the World Health Organization (WHO) [2]. For adults aged 18–64 years, the WHO recommends 150 min at moderate intensity, or 75 min at vigorous intensity. Moreover, the health benefits increase with increased physical activity [2,3]. However, the WHO provides no recommendations for upper limits in terms of intensity, frequency and duration of physical activity exist. Upper limits would help to address a health-related problem: while most people exercise too little, some exercise too much and even display addiction-like behavior [4]. Excessive exercising can have harmful effects on physical (e.g., injury) and mental health (e.g., withdrawal symptoms during absence). Szabo and colleagues defined exercise addiction as “a morbid pattern of behavior in which the habitually exercising individual loses control over his or her exercise habits and acts compulsively, exhibits dependence, and experiences negative consequences to health as well as in his or her social and professional life” [5]. According to the components model by Griffiths, addictions, in general, are characterized by a number of common symptoms, among them withdrawal symptoms, tolerance, mood modification, salience, personal conflict and relapse [6,7,8].

Various studies in previous decades have attempted to clarify the psychological and physiological etiology of exercise dependence [9,10,11]. Synthesizing this research in their review, Berczik and colleagues [4] addressed the necessity of future research into the personal and environmental factors sustaining, maintaining or developing exercise addiction. Susceptibility to addictive behavior in exercise [4] and in other domains has already been associated with factors within a person’s personality, referred to as “personal factors” [12,13], as well as the social environment [14]. Researchers found self-control [13], self-concordant goal setting [12] and social support [14] to be predictive of addictive behavior in other life domains. In the present study, we aim to examine the relationship of self-control, self-concordance (personal factors) and social support (social factor) with exercise dependence.

By suggesting self-concordance as a potential predictor of exercise dependence, we refer to motivation psychology [15]. Sheldon and Elliott (1999) described self-concordance as “the extent to which a goal reflects personal interests and values versus something one feels compelled to do by external or internal pressures [16]. Engaging in self-concordant goals leads to optimal functioning and well-being [15]. Deeper insights to the underlying assumptions can be found in the self-determination theory [17,18]. Attesting to the importance of self-concordance in regard to addictive behaviors, self-concordance is associated with less excessive alcohol consumption [12]. In the same vein, dysfunctional goal characteristics lead to a higher risk of substance abuse and alcoholism [19]. Based on these studies, we assume that self-concordance might also be negatively related to exercise dependence.

Besides the self-concordance of goals [15], self-control is needed to translate goals into action [20]. Self-control is defined as the ability to suppress or control impulses and thereby control one’s own behavior [13,21]. Therefore, persons with stronger self-control can better pursue and succeed in their long-term goals, because they are better at resisting distractions and temptations that might derail goal pursuit. Accordingly, self-control is important for effective self-regulation [22]. Besides the inhibition facet of self-control (e.g., to suppress the urge to eat chocolate in front of the TV), Hoyle and Davisson postulate that the initiation of behavior (e.g., starting to go for a run instead of watching TV), and the continuation of this behavior (e.g., staying with jogging, even when it becomes strenuous) are all facets of the ability of self-control [23].

Studies show that people who can control themselves well have more career success, more stable social relationships and enjoy better physical and mental health [22]. Low self-control, on the other hand, is linked with various individual problems or even social issues, like unhealthy eating, less exercising, impulsive buying, academic failure or underachievement, procrastination, substance abuse or even criminal activities [24,25,26,27,28,29,30,31]. In this study, we examine trait self-control (the relatively stable tendency to exert self-control successfully) as well as state self-control (perception of momentary ability/willingness to apply self-control) as correlates of exercise dependence.

The third variable we assume to be related to exercise dependence is social support. Social support can be provided by a peer group, by friends, or by the family, for example. Social support is defined as “social interactions or relationships that provide individuals with actual assistance or with a feeling of attachment to a person or group that is perceived as loving or caring [32]. Social support is differentiated into perceived social support (perception of the availability of social support) [33] and received social support (the retrospective perception of having been supported) [34].

A large body of evidence shows that social support can contribute to health [35,36,37,38]. Lower mortality rates [39,40], better recovery from surgery [41] and sport injuries [42], conveyance of healthy behavior [43,44], and the enhancement of well-being and motivation [45,46] are just a few examples of these positive effects. Most importantly, in the present research, social support acted as a protective factor for the use of substances or alcohol [47], the frequency of gambling [48] and social media addiction [14]. However, studies analyzing the effects of social support on exercise dependence are scarce. The findings of Lukács and colleagues showed that feelings of not being socially integrated can increase an exerciser’s volume of sport activity [49]. Furthermore, a study with Australian elite athletes from various sports revealed that athletes who are at risk of exercise dependence report lower social support scores compared to their non-at-risk peers [50].

The present research aims to examine personal and social resources simultaneously in order to determine the strength of their potential relationship with exercise dependence. Specifically, we test the hypothesis that athletes with high self-control (trait and state), high exercise-related self-concordance and high levels of social support are less likely to be exercise dependent. Because endurance sports athletes show the greatest risk of exercise dependence [51], we focused on endurance athletes from all types of endurance sports, without any age restrictions except the age of majority in order to increase sample heterogeneity, and therefore the generalizability of our findings. Thus, we extend previous research that focused on specific types of endurance sports [49,52,53,54].

## 2. Materials and Methods

### 2.1. Participants

Two hundred and eleven athletes from different endurance sports took part in an online survey. The subjects were recruited by social media and mailing lists, as well as by written advertisements in sports medicine centers. The inclusion criteria were that athletes had to engage in regular exercise in an endurance sport and had to be a minimum of 18 years old. Due to incomplete data (premature cancellation of the questionnaire), 58 subjects had to be excluded from the study. A further 13 athletes were excluded because they did not meet the inclusion criteria. Finally, the data of 82 male and 58 female (*N* = 140) endurance athletes aged between 18 and 76 years (*M* = 36.35, *SD* = 14.66) were analyzed. Most of the athletes were runners (*n* = 67), triathletes (*n* = 29), and mixed (*n* = 24, for example, running and swimming). Swimming (*n* = 9), cycling (*n* = 10) and rowing (*n* = 1) were less represented in the sample. More detailed descriptions of the sample are presented in the results section.

### 2.2. Procedure

Participants completed a web survey that contained informed consent questions with regard to their sporting activity, as well as the frequency and intensity of the sport, and questionnaires assessing the relevant variables for this research (exercise dependence, self-control, self-concordance, and social support; see below). Participants filled in further questionnaires, which will not be discussed in this paper as they do not relate to the present research question. The web survey ended with a detailed debrief and the information that participants could receive feedback about their scores, as well as some additional information. If they were interested in receiving feedback, they subscribed with their email address, which was saved using a web survey separate from the data provided by the participants. The study was carried out in accordance with the guidelines that were laid out in the Declaration of Helsinki in 1975. Moreover, according to the guidelines of the ethics committee of the first author’s university, no separate Institutional Review Board statement was required for this study. Participants who entered the online study were informed about the purpose of the study, delivered informed consent and confirmed that they voluntarily agreed to participate. In data collection and data processing we followed the compliance of the Health Insurance Portability and Accountability Act (HIPAA).

### 2.3. Measures

Participants indicated their sex, age, occupational status, the frequency and duration of their training sessions, and whether they participate in competitions or not.

In order to assess addictive behavior, the Exercise Dependence Scale (EDS) was used [55]. The scale is based on the Diagnostic and Statistical Manual of Mental Disorders-IV criteria for substance dependence [56]. Hausenblas and Downs used these criteria to operationalize their multidimensional questionnaire for maladaptive behavior in exercising. The athletes were asked to rate their exercise beliefs and behaviors that have occurred in the past three months. Each of the seven dimensions, tolerance (“I continually increase my exercise duration to achieve the desired effects/benefits.”), withdrawal (“I exercise to avoid feeling tense.”), intention effects (“I exercise longer than I intend.”), lack of control (“I am unable to reduce how often I exercise.”), time (“I spend most of my free time exercising.”), reduction in other activities (“I would rather exercise than spend time with family/friends.”) and continuance (“I exercise when injured.”) each had three items with a rating scale from 1 (never) to 6 (always). Average scores for each dimension as well as an overall score were calculated. The EDS allows us to categorize responses in the following manner: (1) at risk—this category includes individuals whose responses are in the “at risk” range, which corresponds to a Likert-scale score of five or six for at least three of the seven criteria; (2) nondependent-symptomatic—this category includes people for whom at least three of the seven criteria are in the “nondependent-symptomatic” range (on the Likert scale, these are the values three and four); and (3) nondependent-asymptomatic—people who are neither addicted nor show symptoms of a sports addiction (on the Likert scale, values of one or two can be found here). Cronbach´s alpha for the overall scale showed an internal consistency of α = 0.88.

Perceived and received social support were measured with the Berlin Social Support Scale [57]. While the perceived scale with eight items measures a prospective (“When everything becomes too much for me to handle, others are there to help me.”), the received scale with 15 items measures a retrospective perspective (“This person made me feel valued and important.”) of social support. Participants rated each statement using a four-point scale ranging from 1 (not at all) to 4 (exactly true). In the present study, the scales showed a high internal consistency (perceived α = 0.94, received α = 0.90).

The Capacity for Self-Control Scale (CSCS) was used to measure the trait dimension of self-control [23]. The scale consists of 20 items that have to be answered on a five-point Likert-scale from 1 (hardly ever) to 5 (nearly always). Examples for items are “I have trouble resisting my cravings.” and “I get started on new projects right away”. Cronbach´s alpha was α = 0.86.

Momentary available self-control was measured using the 25-item State Self-Control Capacity Scale (α = 0.94) [58]. Participants indicated their agreement with statements (e.g., “Example”) using a seven-point rating scale ranging from 1 (not correct at all) to 7 (fully correct). In both questionnaires for self-control the athletes were made aware that the statements did not only refer to sport, but more to life in general.

To measure the self-concordance of participants´ sport- and exercise-related goals, the Self-Concordance in Sport Scale was used [59]. The scale is based on Deci and Ryan´s self-determination theory as well on Sheldon and Elliot´s self-concordance model [15,17]. Participants were asked to rate their intention to exercise in the next few weeks on a six-point Likert scale ranging from 1 (does not apply at all) to 6 (fully applies). Therefore, they had to complete the sentence “In the next few weeks and months, I intend to exercise …”. Intrinsic (“...because I enjoy exercising”), identified (“…by personal decision”), introjected (“…I feel guilty, when I don´t exercise”) and extrinsic (“…I exercise because other people say I should”) forms of regulation were assessed with three items for each subscale. An overall self-concordance score was created by summing the identified and intrinsic scores and then subtracting the introjected and external scores. The internal consistency for the four subscales ranged from α = 0.60 to α = 0.73.

### 2.4. Data Analysis

In all analyses, the assumption of normal distribution was tested with the Shapiro–Wilk test and variance homogeneity was tested with the Levene test. In case of significant main effects, Bonferroni-corrected post hoc tests were calculated to compare differences between specific factor levels. For all tests, statistical significance was set at *p* < 0.05. Cohen´s ds were calculated as effect size estimates, where d > 0.20 represents a small, d > 0.50, moderate, and d > 0.80 a large effect respectively *r* > 0.10 represents a small, *r* > 0.30, moderate, and *r* > 0.50 a large effect at the correlation analyses [60].

In order to test for differences between risk groups in workload and state self-control, one-way analyses of variance (ANOVA) were run. We used T-Tests or Mann–Whitney-U-tests (if normal distribution was violated) to analyze whether men and women differ in the measured variables.

Assessing our hypotheses, correlations between variables were calculated via Pearson correlation coefficients, if the data in the investigated parameters were normal distributed. Otherwise, Spearman correlations were computed. Subsequently, a hierarchical multiple regression with exercise dependence as dependent variable was calculated. At first, the trait factors, self-concordance and trait self-control, were included in the regression equation. As a second step, state self-control was added. For the supplemental analyses, we also conducted a mediation analysis according to the procedure suggested by Baron and Kenny [61]. All analyses were run with JASP, Version 0.14.1, [62] and the statistical software environment R [63]. The plots were created with GGPLOT 2 [64].

## 3. Results

### 3.1. Characteristics of the Sample

Besides the overall score for exercise dependence, we classified athletes into three different risk groups (at risk, nondependent symptomatic, and nondependent asymptomatic) according to a classification system proposed by Hausenblas and Downs [55]. Eight athletes were identified as “at risk” for exercise dependence while 93 athletes could be classified as “nondependent symptomatic” and 39 athletes as “nondependent asymptomatic”.

**Figure 1:**
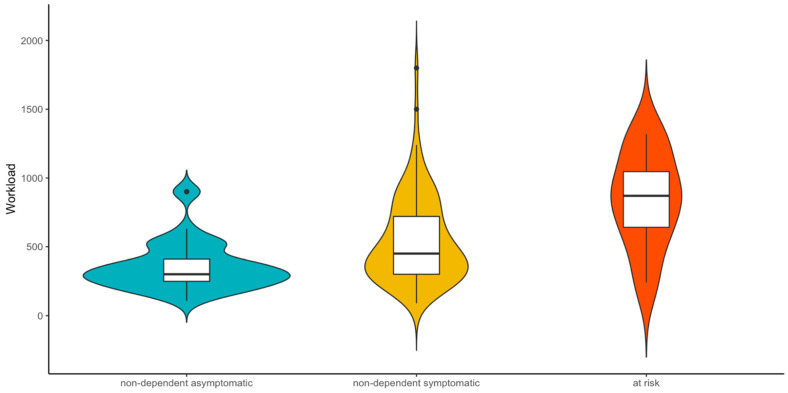
Workload per week (minutes × unit) illustrated for the three classifications of exercise dependence.

On average, the athletes had been involved with their sports for 12.77 years (*SD* = 9.25) and reported that they exercise *M* = 6.01 (*SD* = 3.18) times per week for *M* = 85.46 min (*SD* = 33.45) per session. This results in an average workload of *M* = 512.23 min (*SD* = 305.88) per week. Eleven participants reported that they do not take part in competitions. Furthermore, seven athletes train with a coach, while 83 exercise alone and 50 practice in a group. Related to the classification of exercise dependence, the “at risk” group exercises for 110 min (*SD* = 58.80) per training session and exercises more often (*M_units_* = 8.31, *SD* = 3.72) than the nondependent symptomatic (*M_minutes_* = 86.71, *SD* = 30.59; *M_units_* = 6.37, *SD* = 3.42) and the nondependent asymptomatic groups (*M_minutes_* = 77.42, SD = 31.52; *M_unit_*_s_ = 4.69, *SD* = 1.70). In order to test the differences between workload, a one-way ANOVA was run. Levene´s test showed a significant result, so the analysis was corrected with the Welch test. The analysis revealed a significant main effect, *F*(2, 18.54) = 14.26, *p* < 0.001, *η*^2^ = 0.15. Post hoc tests with Bonferroni correction showed that athletes in the “at risk” group showed a significantly higher workload (*M* = 840.00 min per week, *SD* = 353.42) compared to athletes in the “nondependent symptomatic” group (*M* = 548.89 min per week, *SD* = 312.95), *t* = −2.77, *p* = 0.019, *d* = −0.92, and the athletes in the nondependent asymptomatic group (*M* = 357.58 min per week, *SD* = 177.95), *t* = −4.37, *p* < 0.001, *d* = −2.25. Furthermore, athletes in the “nondependent symptomatic” group have a significantly higher workload than persons with no symptoms, *t* = −3.53, *p* = 0.002, *d* = −0.68. The different workloads are illustrated in Figure 1.

### 3.2. Preliminary Analysis

In their review, Dumitru and colleagues [65] examined gender differences in the prevalence of exercise dependence and concluded that men are more at risk for exercise addiction than women. Therefore, we tested for gender differences in exercise dependence and in the other dependent variables. Table 1 shows the results of t-tests (for normally distributed variables) and Mann–Whitney U-tests (for non-normally distributed variables). The analyses revealed a significant difference for state self-control with higher scores for men (*M* = 5.27, *SD* = 1.00) compared to women (*M* = 4.92, *SD* = 1.04), *W* = 2842, *p* = 0.050, *d* = 0.20.

### 3.3. Descriptive Statistics and Correlation Analyses

Pearson correlations and Spearman correlations (if nonparametric test is required) were computed between trait and state self-control, self-concordance, perceived and received social support and exercise dependence (see Table 2). Small to moderate negative correlations were observed between exercise dependence and self-concordance (*r* = −0.23, *p* = 0.006), trait self-control (*r* = −0.26, *p* = 0.002) and state self-control (*r* = −0.39, *p* < 0.001). In contrast, exercise dependence was not significantly related to social support.

Moderate to large positive correlations were found between self-concordance and trait self-control (*r* = 0.36, *p* < 0.001) and self-concordance and state self-control (*r* = 0.53, *p* < 0.001). Furthermore, a large positive correlation of trait and state self-control was observed (*r* = 0.49, *p* < 0.001). Self-concordance and social support were positively correlated (perceived: *r* = 0.21, *p* = 0.002; received: *r* = 0.18, *p* = 0.038). State and trait self-control were positively correlated to perceived social support (trait: *r* = 0.21, *p* = 0.008; state: *r* = 0.29, *p* < 0.001).

### 3.4. Regression Analyses

The correlation analyses showed that social support (social factor) is not related to exercise dependence, whereas all three personal factors revealed a negative relationship with exercise dependence. To further explore the strength of the effects and disentangle the effects of trait and state effects, a hierarchical multiple regression was computed. At first, the trait factors, self-concordance and trait self-control, were included in the regression equation (Model 1). Then, as a second step, state self-control was added in Model 2.

The results of these regression analyses are summarized in Table 3. Model 1 was significant, *F*(2, 137) = 5.84, *p* = 0.004, but neither self-concordance, nor trait self-control were revealed as significant predictors. However, their effects were marginally significant at *p* < 0.10, explaining why Model 1 was significant. When accounting for state self-control (Model 2), the trait factors became nonsignificant (see Table 3) and state self-control was the only significant statistical predictor of exercise dependence. Overall, Model 2 was significant, *F*(3, 136) = 8.54, *p* = 0.001.

### 3.5. Mediation Analyses—Self-Control, Self-Concordance and Exercise Dependence

**Figure 2:**
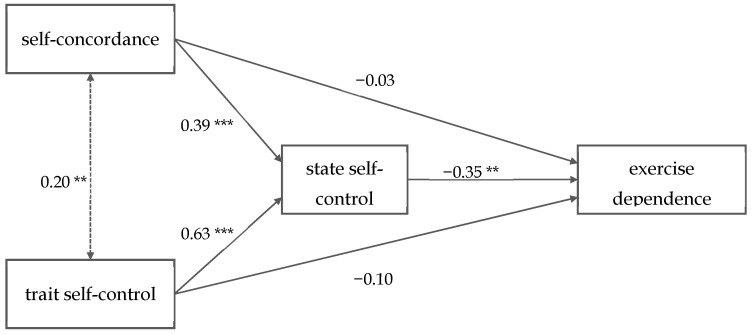
The pathways of the mediation model. Coefficients are unstandardized. ** *p* < 0.01, *** *p* < 0.001.

The results of the correlational pattern between variables suggest that the effects of self-concordance and trait self-control on exercise dependence could be mediated by state self-control. A mediation analysis including both predictors (see Figure 2) showed that the relationship between self-concordance and exercise dependence (indirect effect: z = −2.99, *se* = 0.05, *p* = 0.003, 95% CI [−0.227, −0.047], total effect: z = −1.88, *se* = 0.09, *p* = 0.061, 95% CI [−0.342, 0.007]) as well as the relationship between trait self-control and dependence (indirect effect: z = −2.87, *se* = 0.08, *p* = 0.004, 95% CI [−0.375, −0.070], total effect: z = −1.98, *se* = 0.16, *p* = 0.048, 95% CI [−0.643, −0.003]), were fully mediated by state self-control. An overview of the unstandardized model parameters is given in Figure 2.

### 3.6. Supplemental Analysis—Risk Factor Groups

The linear regression analyses revealed that momentary available self-control predicts exercise dependence. A one-way ANOVA testing whether risk factor groups differed in state self-control was significant *F*(2, 18.65) = 7.60, *p* = 0.004, *η*^2^ = 0.10. A Bonferroni-corrected post hoc test revealed that the nondependent asymptomatic group (*M* = 5.58, *SD* = 0.96) scored significantly higher score on state self-control than the nondependent symptomatic (*M* = 5.01, *SD* = 0.99; *t* = 3.06, *p* = 0.008, *d* = 0.59), and the at-risk groups (*M* = 4.26, *SD* = 1.03; *t* = 3.47, *p* = 0.002, *d* = 1.36). There was no significant difference between the nondependent symptomatic and at-risk groups (*t* = 2.07, *p* = 0.122, *d* = 0.76).

## 4. Discussion

The aim of this study was to promote research in exercise dependence by taking personal factors from motivational research (self-concordance) and volitional research (self-control), as well as social support, into account simultaneously. In support for our hypotheses, self-control and self-concordance were negatively related to exercise dependence. Further unpacking this relationship, we found that state self-control mediated the link between trait self-control, self-concordance and exercise dependence. Trait self-control and self-concordance of exercise goals seem to activate high momentary available self-control, which, in turn, was associated with a reduced risk of exercise dependence. This is in line with prior work by Sheldon and Elliot [15], which found that self-concordant goals lead to sustained effort, which, in turn, led to better goal attainment and well-being. In the context of the present study, high self-concordance might facilitate heightened perceptions of momentary self-control (triggering higher effort), which might protect against detrimental behavioral tendencies (e.g., excessive exercise). The perception of higher momentary self-control might result from the fact that striving for goals that are *not* self-concordant requires additional effort, while current theorizing on self-control indicates that the exertion of self-control reduces the willingness to invest further effort [66]. In contrast, self-concordant goals themselves require less effort, which could, in turn, be invested into successful task execution.

The findings from this study reveal that trait and state self-control are linked to each other in predicting the risk of addictive behavior in exercising. Several approaches regarding how trait self-control might elucidate state self-control can be found in the literature (for an overview, see de Ridder, 2018) [67]. First, besides the assumption that successfully dealing with self-control dilemmas can be traced back to successful inhibition, a meta-analysis from de Ridder and colleagues [22] emphasizes that individuals with high trait self-control reported stronger automatized adaptive behavior like habits or routines that can be performed without effort. Second, they organize themselves and their environment in a way that means they need to deal with effortful inhibitions to a lesser extent in everyday life because this structure prevents them from being constantly confronted with problematic temptations [68]. This is also in line with Duckworth´s considerations on situational self-control [69].

According to Ent et al., 2015, self-control could be interpreted as a proactive trait that is helpful avoiding harmful behaviors [70]. In terms of our study context, it is likely that athletes with high trait self-control structure their lives in a more adaptive way, and that maintenance of their lives is less demanding in terms of the application of constant self-control. Nevertheless, once a self-control dilemma occurs, these individuals are able to deal with this dilemma more efficiently [71]. In relation to the expression of the exercise dependence scale in terms of continuance, for example, a dilemma could be that an athlete striving for an improvement in their personal best in a half marathon is interrupted by an injury. In order to reach this goal, regular exercising is necessary. Therefore, the athlete’s aim to improve is in conflict with the need to recover from the injury. Thus, an athlete with high state self-control would be able to make a better choice when an injury occurred, as further exercising would be harmful to their health.

Furthermore, the supplemental analysis on the group level supports our findings from the linear analysis. Athletes in the “nondependent asymptomatic” group showed significantly higher momentary self-control than the “nondependent symptomatic and at risk” group.

In our sample, 7% of endurance sport athletes belonging to the “at risk” group, 66% to the “nondependent symptomatic” group and 28% to the “nondependent asymptomatic” group. Previous studies display similar distributions for the risk of exercise dependence in their samples [49,51,72,73,74]. Moreover, comparing the three groups “at risk”, “nondependent symptomatic”, and “nondependent asymptomatic” shows that the different workloads in the groups can be traced back to the fact that athletes in these groups attend more training sessions per week rather than to the duration of a session. This finding is supported by studies that revealed a positive relationship between the total duration spent training and the risk of exercise dependence [75,76].

Contrary to our assumptions, neither perceived nor received support is directly related to exercise dependence in this study. Moreover, unexpectedly, social support was positively related to self-concordance and self-control, and is therefore associated with facets of self-regulation. Why social support does not affect a specific domain of self-regulation (exercise dependence) remains an unanswered question. We can only speculate that the different levels of abstraction of the concepts (general: self-control, self-concordance; sport-specific: dependence) might provide a rationale that should be tested empirically.

There are some limitations to this study that should be acknowledged. Our study is cross-sectional. Therefore, the line of reasoning in mediation analysis is based on theoretical deliberations and cannot take temporal sequencing of variables into account. Furthermore, estimated mediation effects are based on correlational relations between the variables, which does not allow for any specifications about directionality. Deeper insights and advanced interpretations of directions and causality of effects would be enabled by longitudinal data, especially since developing an exercise dependence is likely to develop gradually and over time. In consideration of the fluctuation of personal state factors in contrast to stable personal trait factors (e.g., self-control) [66], a longitudinal design could further lead to a better understanding of the observed relation between state and trait factors predicting the risk of harmful exercising.

Although our study extends the current research on exercise dependence by simultaneously taking motivational and volitional factors into account, our study is limited in its design. We used a self-report questionnaire (EDS), which is based on a risk score and risk categorization (at risk, nondependent symptomatic and nondependent asymptomatic) and is not a clinical diagnosis instrument for addiction. However, it should be mentioned that exercise dependence is not classified within any medical or psychological diagnostic frameworks [4]. Moreover, the interpretation of self-reported data is complicated because of possible bias due to the nature of the studied sample (e.g., demographics, gender and race/ethnicity). Szabo and colleagues suggested that future research in the field of exercise dependence should be extended with in-depth interviews with athletes suspected to be at risk of exercise dependence by those instruments [5]. This kind of mixed-methods approach, using quantitative as well as qualitative data, could lead to a better understanding and characterization of exercise dependence and its symptoms, as well as its promoting and preventing factors (like self-control and self-concordance). Once these mechanisms become clearer, it will be easier to explore possible prevention strategies and recommend appropriate interventions.

Practical implications can be derived from our findings that address the three components in our mediation model (see Figure 2). According to self-concordance, athletes and coaches were recommend spending effort to choose goals that fit the values, interests, and personality of the athlete. As reported by Sheldon, knowing one’s implicit motivations and potentials are important for setting more self-concordant goals. This can be reached by interpersonal contexts (e.g., coach–athlete relationships) that promote accurate self-insights and provide autonomy support [77]. Moreover, Weinberg and Gould provide a good overview of implementation strategies of goal setting and explain the underlying key processes [78].

Self-control, as the second component of our mediation model, can be improved by specific self-control training (for a review of self-control training effects see Friese and colleagues) [79]. These trainings are theoretically based on the assumption of the strength model of self-control that self-control can be trained as a muscle [80]. Repeated exposure to self-control (for example by pressing a hand-grip device regularly or simply brushing one´s teeth with the non-dominant hand) build up self-control [81].

The third component, state self-control, can be modified by reducing other tasks that require self-control (see ego depletion effect) [21,80,82]. A simple example would be to reduce stress at work or to better structure daily life and environment.

## 5. Conclusions

Our results showed that self-control and self-concordance play a key role in predicting exercise dependence in endurance athletes, whereas, unexpectedly, social support did not explain the variance in exercise dependence. Additionally, the present study suggests that both trait factors decrease the risk of exercise dependence by increasing perceptions of momentary self-control. These findings can help to enhance awareness when dealing with high training volumes and the development of addictive exercising behavior in endurance sports at recreational and elite levels. Mixed-methods procedures (e.g., also including lab experiments, in that self-concordance of an exercise goal is experimentally manipulated) and longitudinal studies are recommended to shed further light on the assumed mechanism.

## Figures and Tables

**Table 1:** Descriptive statistics and tests for differences between male and female athletes.

	Total (*N* = 140)	Male (*n* = 82)	Female (*n* = 58)	*t*/*U*	*p*
	*M* (*SD*)	*M* (*SD*)	*M* (*SD*)		
Exercise dependence	2.98 (0.70)	2.94 (0.73)	3.04 (0.65)	−0.85	0.395
Self-control (trait)	3.52 (0.54)	3.49 (0.58)	3.56 (0.47)	−0.76	0.446
Self-concordance *	3.16 (0.98)	3.15 (0.92)	3.17 (1.08)	2311.50	0.780
Self-control (state) *	5.13 (1.03)	5.27 (1.00)	4.92 (1.04)	2842.00	0.050
Perceived support *	3.61 (0.51)	3.56 (0.55)	3.69 (0.45)	1986.00	0.087
Received support *	3.33 (0.54)	3.28 (0.57)	3.39 (0.50)	2117.50	0.271
Workload *	512.16 (305.88)	549.54 (320.39)	459.48 (278.30)	2814.50	0.065

Note. * Assumption of normal distribution was violated. Therefore, a non-parametric test was used.

**Table 2:** Correlations and descriptive statistics for the dependent variables.

Variables	EDS	SCT	SSC	SCS	PER	REC	WKL
Exercise dependence—EDS	1						
Self-control (trait)—SCT	−0.26 **	1					
Self-concordance—SSC	−0.23 **	0.36 ***	1				
Self-control (state)—SCS	−0.39 ***	0.49 ***	0.53 ***	1			
Perceived support—PER	−0.10	0.21 *	0.21 *	0.29 ***	1		
Received support—REC	0.01	0.18 *	0.18 *	0.14	0.52 ***	1	
Workload—WKL	0.31 ***	0.10	0.05	0.06	−0.01	0.12	1
Mean	3.01	3.50	3.08	5.09	3.59	3.32	526.28
*SD*	0.70	0.55	1.04	1.03	0.54	0.54	334.11
Range	1–7	1–5	−6–6	1–7	1–4	1–4	90–1800

Note 2. The Pearson correlation was run for the Exercise Dependence Scale (EDS) and self-concordance (SSC), for EDS and self-control (state) (SCS) and for SSC and SCS. For all other analyses, Spearman’s Rho was calculated. * *p* < 0.05, ** *p* < 0.01, *** *p* < 0.001.

**Table 3:** Regression analyses predicting exercise dependence.

Variable	Model 1					Model 2				
	*b*	*SE b*	*β*	*p*	95% CI	*b*	*SE b*	*β*	*p*	95% CI
Intercept	4.14	0.38		0.001		4.52	0.38		0.001	
Self-concordance	−0.12	0.06	−0.16	0.066	[−0.241, 0.008]	−0.02	0.07	−0.03	0.751	[−0.151, 0.109]
Trait self-control	−0.23	0.12	−0.18	0.052	[−0.453, 0.002]	−0.07	0.12	−0.05	0.556	[−0.305, 0.165]
State self-control						−0.24	0.07	−0.35	0.001	[−0.371, −0.108]
R^2^	0.08					0.16				
Adj. R^2^	0.07					0.14				

Note. SE: standard error. CI = Confidence interval.

## Data Availability

Data will be provided by the authors upon request.
